# Supplementary material for: Dynamic transcriptomic profiles of zebrafish gills in response to zinc supplementation
Source: BMC Genomics. 2010 Oct 11;11:553. doi: 10.1186/1471-2164-11-553 (PMC3091702; doi:10.1186/1471-2164-11-553)
Supplement: Additional file 2 — Interactive Direct Interaction Network representing the molecular interactions between zinc, copper, iron, calcium and proteins encoded by transcripts changed by zinc supplementation. Mini web-site containing index.html and hyperlinked pages in subdirectory describing a Direct Interaction Network automatically generated based on curated interactions contained within the proprietary PathwayArchitect database. Ovals represent proteins and the circles symbolize metal ions. Objects are coloured by their abundance in zebrafish at the time-point they were significantly different from the control is a scale from -4 fold (dark green) to +4 fold (dark red). Where significant differences were found at more than one time-point, the colour overlay shows expression at the first instance. Dark blue squares denote 'binding', and light blue squares 'expression'; green squares stand for 'regulation', green diamonds for 'metabolism', and green circles for 'promoter binding'. Arrow heads indicate directionality of the interaction where annotated. All nodes and edges can be further interrogated by selecting the relative area of the image. [file 1471-2164-11-553-S2.zip › PathwayArchitect Zn xs DIN/636424.html]

# REGULATION:

|  |  |
| --- | --- |
| Type | REGULATION |
| Effect | Positive |


---

|  |  |
| --- | --- |
| Score | 0 |


---

|  |  |
| --- | --- |
| Reference Count | 8 |


---

|  |  |
| --- | --- |
| Mechanism | Unknown |


---

|  |  |
| --- | --- |
| Reference:0 || Sentence | "However, Ca2+ significantly increased the accessibility of the TnI-1 epitope on TnI in the presence of troponin C and troponin T." |
| PMID | 11327886 |
| Year | 2001 |
| Species | Mouse |
|  | Human |
|  | Rat |
| Journal | Biochemistry |
| RefScore | 2 |
| Source | PArchNLP |
  |
|  |  |


---

|  |  |
| --- | --- |
 Reference:1 || Sentence | "The results demonstrate that the splice donor site mutation in the cardiac TnT gene impairs the regulatory function of the TnT molecule, leading to an increase in the Ca(2+) sensitivity, and a decrease in the cooperativity, of cardiac muscle contraction, which might be involved in the pathogenesis of HCM." |
| PMID | 10444398 |
| Year | 1999 |
| Species | Human |
| Journal | Am J Physiol |
| RefScore | 0 |
| Source | PArchNLP |
  ||  |  |


---

|  |  |
| --- | --- |
 Reference:2 || Sentence | "It is shown that the amount of Tn complex formed by TnT1-201 rather than the activity of the mutant Tn complex affected this Ca2+ sensitivity." |
| PMID | 8987992 |
| Year | 1996 |
| Species | Human |
| Journal | Biochemistry |
| RefScore | 2 |
| Source | PArchNLP |
  ||  |  |


---

|  |  |
| --- | --- |
 Reference:3 || Sentence | "Overall, our results also imply that residues 159-201 constitute the smallest region of TnT which contributes to the Ca2+ sensitivity of actoS1 ATPase in a reconstituted regulatory system." |
| PMID | 8987992 |
| Year | 1996 |
| Species | Human |
| Journal | Biochemistry |
| RefScore | 1 |
| Source | PArchNLP |
  ||  |  |


---

|  |  |
| --- | --- |
 Reference:4 || Sentence | "To determine whether the different N-terminal isoforms are functionally distinct with respect to structure, Ca(2+) sensitivity, and inhibition of force development, the four known human cardiac troponin T isoforms, TnT1 (all exons present), TnT2 (missing exon 4), TnT3 (missing exon 5), and TnT4 (missing exons 4 and 5), were expressed, purified, and utilized in skinned fiber studies and in reconstituted actomyosin ATPase assays." |
| PMID | 12093807 |
| Year | 2002 |
| Species | Human |
| Journal | J Biol Chem |
| RefScore | 0 |
| Source | PArchNLP |
  ||  |  |


---

|  |  |
| --- | --- |
 Reference:5 || Sentence | "The Ca(2+) signaling apparatus includes 1) the ryanodine receptor that is the sarcoplasmic reticulum Ca(2+) release channel, 2) the troponin protein complex that mediates the Ca(2+) effect to the myofibrillar structures leading to contraction, 3) the Ca(2+) pump responsible for Ca(2+) reuptake into the sarcoplasmic reticulum, and 4) calsequestrin, the Ca(2+) storage protein in the sarcoplasmic reticulum." |
| PMID | 10893434 |
| Year | 2000 |
| Species | Human |
| Journal | Physiol Rev |
| RefScore | 3 |
| Source | PArchNLP |
  ||  |  |


---

|  |  |
| --- | --- |
 Reference:6 || Sentence | "Exchanging the mutant cTnT into isolated cardiac myofibrils also increased the free Ca(2+) concentrations required for the activation of ATPase." |
| PMID | 11773635 |
| Year | 2002 |
| Species | Human |
| Journal | Proc Natl Acad Sci U S A |
| RefScore | 0 |
| Source | PArchNLP |
  ||  |  |


---

|  |  |
| --- | --- |
 Reference:7 || Sentence | "The results indicate that cTnT mutations found in the two distinct forms of cardiomyopathy (i.e., HCM and DCM) change the Ca(2+) sensitivity of cardiac muscle contraction in opposite directions." |
| PMID | 11773635 |
| Year | 2002 |
| Species | Human |
| Journal | Proc Natl Acad Sci U S A |
| RefScore | 1 |
| Source | PArchNLP |
  |


---

|  |  |
| --- | --- |
